# Correction: Dynamical Hurst analysis identifies EEG channel differences between PTSD and healthy controls

**DOI:** 10.1371/journal.pone.0214527

**Published:** 2019-03-21

**Authors:** Bahareh Rahmani, Chung Ki Wong, Payam Norouzzadeh, Jerzy Bodurka, Brett McKinney

In Table 1, there was an error when importing the data that made the summary calculation wrong. Please see the corrected summary statistics and Table 1 here.

**Table 1:** Summary of EEG statistics for PTSD and healthy subjects for channel F3.

Subject	Mean	Std.	Skewness	Kurtosis
**Healthy**	29.2511	15.7567	-0.4091	4.8623
**PTSD**	32.4275	8.8147	0.2089	2.5751
