# Supplementary material for: Dynamical Hurst analysis identifies EEG channel differences between PTSD and healthy controls
Source: PLoS One. 2018 Jul 3;13(7):e0199144. doi: 10.1371/journal.pone.0199144 (PMC6029761; doi:10.1371/journal.pone.0199144)
Supplement: S1 Appendix — Zip file containing files: Standard Deviations of Healthy 1 - H3_w3.txtStandard Deviations of Healthy 2 - H4_w3.txtStandard Deviations of Healthy 3 - H5_w3.txtStandard Deviations of Healthy 4 - H6_w3.txtStandard Deviations of Healthy 5 - H7_w3.txtStandard Deviations of Healthy 6 - H12_w3.txtStandard Deviations of PTSD 1 - P1_w3.txtStandard Deviations of PTSD 2 - P2_w3.txtStandard Deviations of PTSD 3 - P8_w3.txtStandard Deviations of PTSD 4 - P9_w3.txtStandard Deviations of PTSD 5 - P10_w3.txtStandard Deviations of PTSD 6 - P11_w3.txtHurst Values of Healthy 1 - Hurst_H3.txtHurst Values of Healthy 2 - Hurst_H4.txtHurst Values of Healthy 3 - Hurst_H5.txtHurst Values of Healthy 4 - Hurst_H6.txtHurst Values of Healthy 5 - Hurst_H7.txtHurst Values of Healthy 6 - Hurst_H12.txtHurst Values of PTSD 1 - Hurst_P1.txtHurst Values of PTSD 2 - Hurst_P2.txtHurst Values of PTSD 3 - Hurst_P8.txtHurst Values of PTSD 4 - Hurst_P9.txtHurst Values of PTSD 5 - Hurst_P10.txtHurst Values of PTSD 6 - Hurst_P11.txtOriginal Data Healthy 1 - H3.txtOriginal Data Healthy 2 - H4.txtOriginal Data Healthy 3 - H5.txtOriginal Data Healthy 4 - H6.txtOriginal Data Healthy 5 - H7.txtOriginal Data Healthy 6 - H12.txtOriginal Data PTSD 1 - P1.txtOriginal Data PTSD 2 - P2.txtOriginal Data PTSD 3 - P8.txtOriginal Data PTSD 4 - P9.txtOriginal Data PTSD 5 - P10.txtOriginal Data PTSD 6 - P11.txt. (ZIP) [file pone.0199144.s001.zip › Appendix.docx]

**Supplemental Material**

**Dynamical Hurst Analysis Identifies EEG Channel**

**Differences Between PTSD and Healthy Controls**

Bahareh Rahmani^1,2,^*, Chung Ki Wong^3^, Payam Norouzzadeh^4^, Jerzy Bodurka^3,5^, Brett McKinney^1^

^1^Tandy School of Computer Science and Department of Mathematics, University of Tulsa, Tulsa, OK, USA

^2^Mathematics and Computer Science Department, Fontbonne University, Saint Louis, MO, USA

^3^Laureate Institute for Brain Research (LIBR), Tulsa, OK, USA

^4^Helmerich Advanced Technology Research Center, Oklahoma State University, Tulsa, Oklahoma, USA

^5^Stephenson School of Biomedical Engineering, University of Oklahoma, Tulsa, OK, USA

**APPENDIX I:** Box plot of original data. Vertical: EEG data points (µV). Horizontal: subjects – H: healthy controls, P: PTSD cases

EEG data (µV)

EEG data (µV)

EEG data (µV)

EEG data (µV)

EEG data (µV)

EEG data (µV)

EEG data (µV)

EEG data (µV)

EEG data (µV)

EEG data (µV)

EEG data (µV)

EEG data (µV)

EEG data (µV)

EEG data (µV)

EEG data (µV)

EEG data (µV)

EEG data (µV)

EEG data (µV)

EEG data (µV)

EEG data (µV)

EEG data (µV)

EEG data (µV)

EEG data (µV)

EEG data (µV)

EEG data (µV)

EEG data (µV)

EEG data (µV)

EEG data (µV)

EEG data (µV)

EEG data (µV)

EEG data (µV)

**APPENDIX II:** Standard deviation in the preliminary analysis using 8 subjects to find the stationary points – blue: healthy controls, red: PTSD cases

**
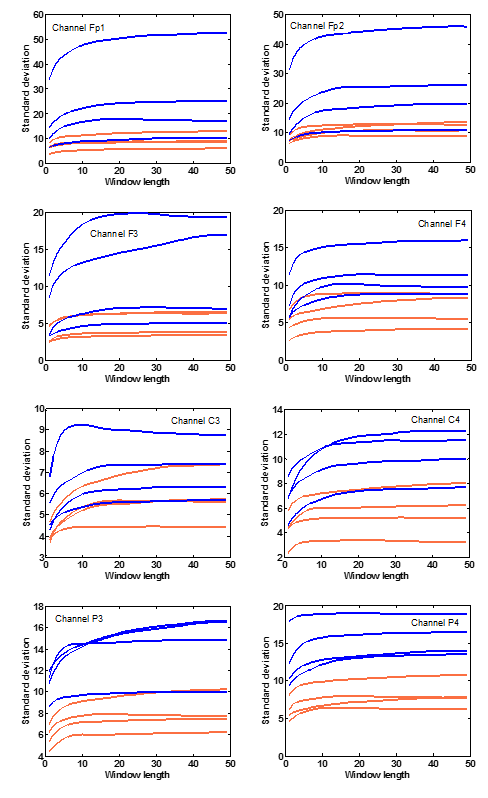
**

**
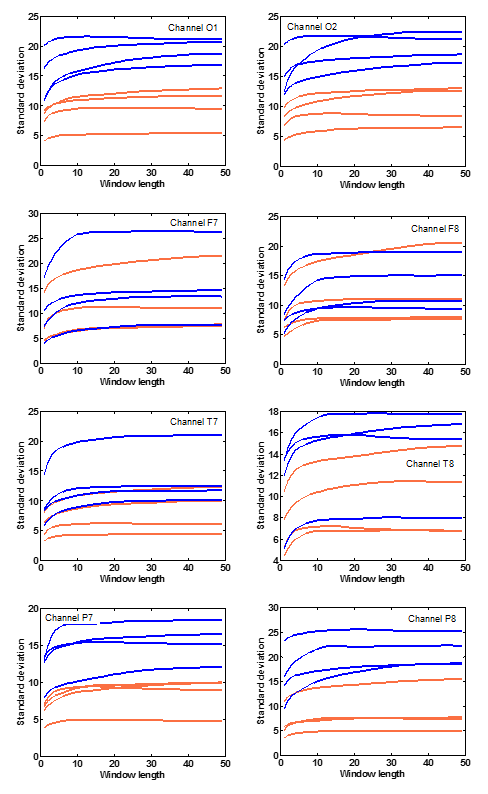
**

**-**

**
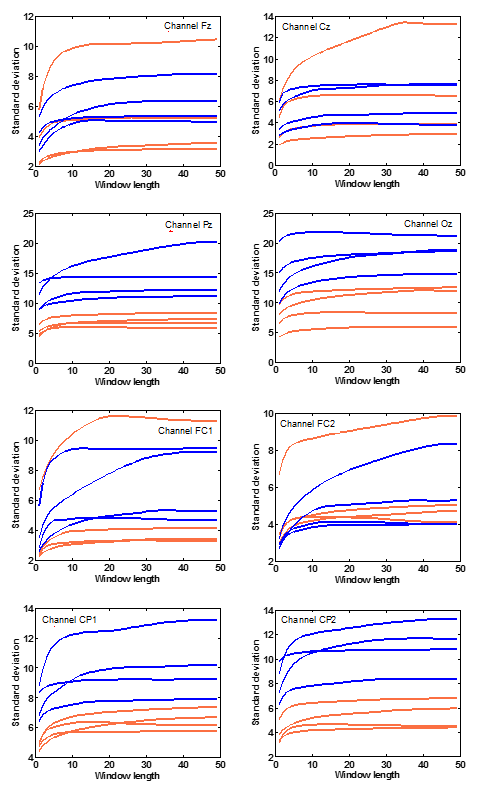
**

**
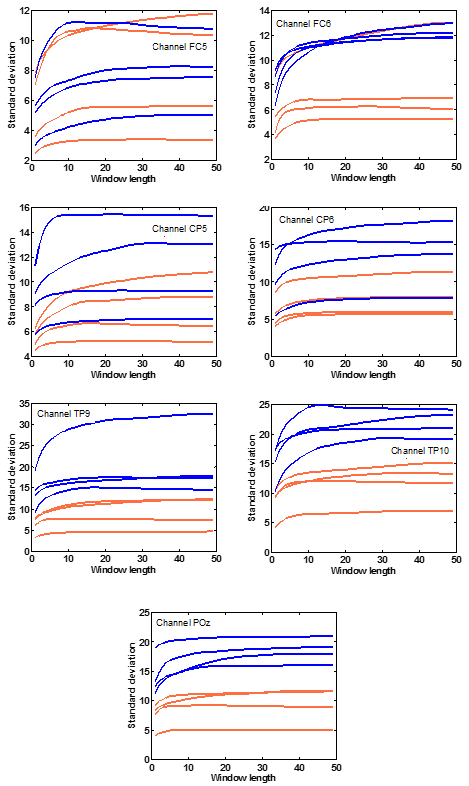
**

**APPENDIX III:** Hurst exponents in the preliminary analysis using 8 subjects– blue: healthy controls, red: PTSD cases.

**
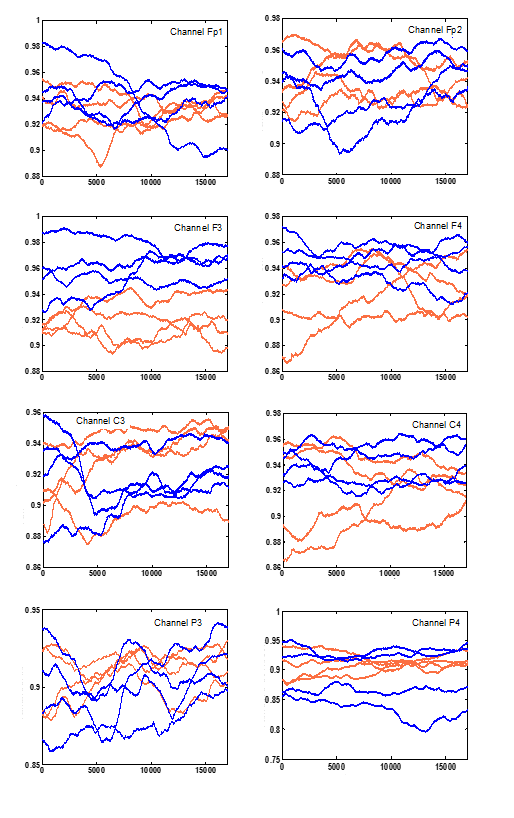
**

Hurst Exponent

Hurst Exponent

Window Number

Window Number

Hurst Exponent

Hurst Exponent

Window Number

Window Number

Hurst Exponent

Hurst Exponent

Hurst Exponent

Hurst Exponent

Window Number

Window Number

Window Number

Window Number


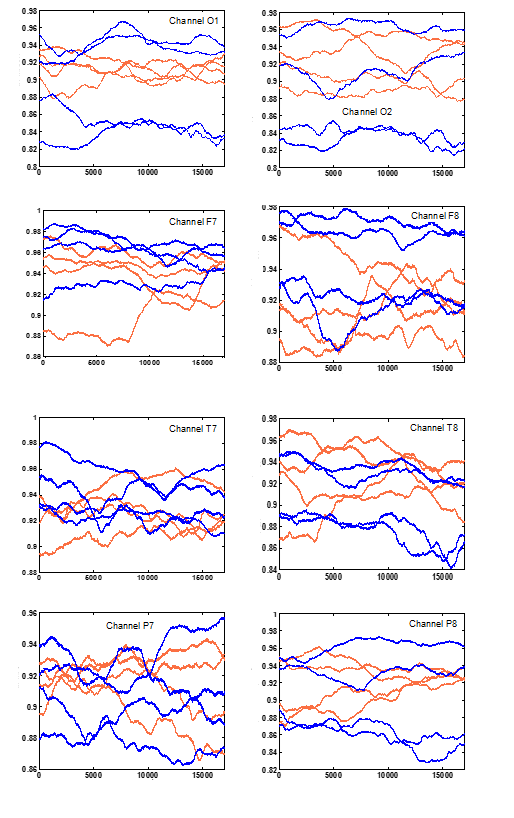


Hurst Exponent

Hurst Exponent

Hurst Exponent

Hurst Exponent

Hurst Exponent

Hurst Exponent

Hurst Exponent

Hurst Exponent

Window Number

Window Number

Window Number

Window Number

Window Number

Window Number

Window Number

Window Number

**
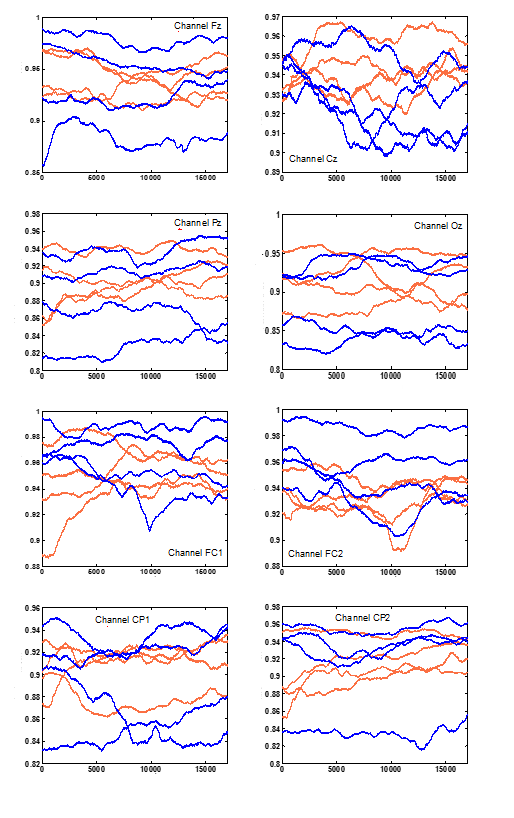
**

Hurst Exponent

Hurst Exponent

Hurst Exponent

Hurst Exponent

Hurst Exponent

Hurst Exponent

Hurst Exponent

Hurst Exponent

Window Number

Window Number

Window Number

Window Number

Window Number

Window Number

Window Number

Window Number

**
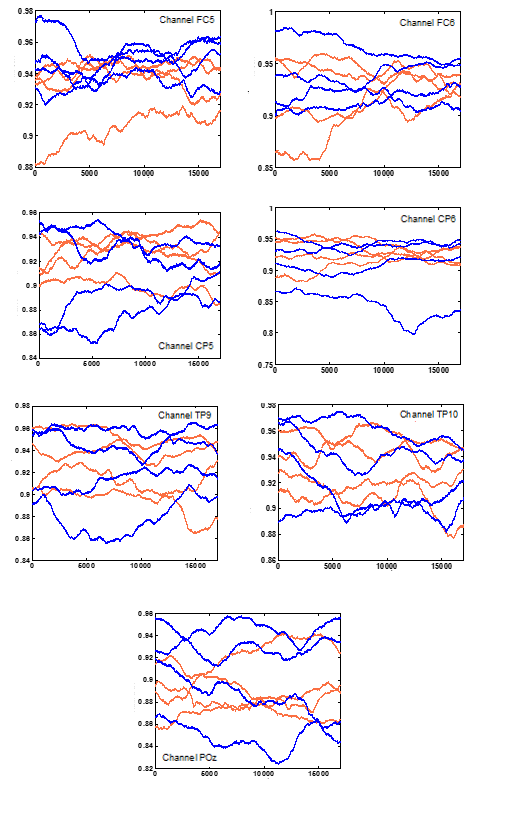
**

Hurst Exponent

Hurst Exponent

Hurst Exponent

Hurst Exponent

Hurst Exponent

Hurst Exponent

Hurst Exponent

Window Number

Window Number

Window Number

Window Number

Window Number

Window Number

Window Number
