# Supplementary material for: Dynamical Hurst analysis identifies EEG channel differences between PTSD and healthy controls
Source: PLoS One. 2018 Jul 3;13(7):e0199144. doi: 10.1371/journal.pone.0199144 (PMC6029761; doi:10.1371/journal.pone.0199144)
Supplement: S1 Appendix — Zip file containing files: Standard Deviations of Healthy 1 - H3_w3.txtStandard Deviations of Healthy 2 - H4_w3.txtStandard Deviations of Healthy 3 - H5_w3.txtStandard Deviations of Healthy 4 - H6_w3.txtStandard Deviations of Healthy 5 - H7_w3.txtStandard Deviations of Healthy 6 - H12_w3.txtStandard Deviations of PTSD 1 - P1_w3.txtStandard Deviations of PTSD 2 - P2_w3.txtStandard Deviations of PTSD 3 - P8_w3.txtStandard Deviations of PTSD 4 - P9_w3.txtStandard Deviations of PTSD 5 - P10_w3.txtStandard Deviations of PTSD 6 - P11_w3.txtHurst Values of Healthy 1 - Hurst_H3.txtHurst Values of Healthy 2 - Hurst_H4.txtHurst Values of Healthy 3 - Hurst_H5.txtHurst Values of Healthy 4 - Hurst_H6.txtHurst Values of Healthy 5 - Hurst_H7.txtHurst Values of Healthy 6 - Hurst_H12.txtHurst Values of PTSD 1 - Hurst_P1.txtHurst Values of PTSD 2 - Hurst_P2.txtHurst Values of PTSD 3 - Hurst_P8.txtHurst Values of PTSD 4 - Hurst_P9.txtHurst Values of PTSD 5 - Hurst_P10.txtHurst Values of PTSD 6 - Hurst_P11.txtOriginal Data Healthy 1 - H3.txtOriginal Data Healthy 2 - H4.txtOriginal Data Healthy 3 - H5.txtOriginal Data Healthy 4 - H6.txtOriginal Data Healthy 5 - H7.txtOriginal Data Healthy 6 - H12.txtOriginal Data PTSD 1 - P1.txtOriginal Data PTSD 2 - P2.txtOriginal Data PTSD 3 - P8.txtOriginal Data PTSD 4 - P9.txtOriginal Data PTSD 5 - P10.txtOriginal Data PTSD 6 - P11.txt. (ZIP) [file pone.0199144.s001.zip › Marked_Manuscript.docx]

**Dynamical Hurst Analysis Identifies EEG Channel**

**Differences Between PTSD and Healthy Controls**

Bahareh Rahmani^1,2,*^, Chung Ki Wong^3^, Payam Norouzzadeh^4^, Jerzy Bodurka^3,5^, Brett McKinney^1^

^1^Tandy School of Computer Science and Department of Mathematics, University of Tulsa, Tulsa, OK, USA

^2^Mathematics and Computer Science Department, Fontbonne University, Saint Louis, MO, USA

^3^Laureate Institute for Brain Research (LIBR), Tulsa, OK, USA

^4^Helmerich Advanced Technology Research Center, Oklahoma State University, Tulsa, Oklahoma, USA

^5^Stephenson School of Biomedical Engineering, University of Oklahoma, Tulsa, OK, USA

* Corresponding author

E-mail: Brahmani@fontbonne.edu

**Abstract**

We employ a time-dependent Hurst analysis to identify EEG signals that differentiate between healthy controls and combat-related PTSD subjects. The Hurst exponents, calculated using a rescaled range analysis, demonstrate a significant differential response between healthy and PTSD samples which may lead to diagnostic applications. To overcome the non-stationarity of EEG data, we apply an appropriate window length wherein the EEG data displays stationary behavior. We then use the Hurst exponents for each channel as hypothesis test statistics to identify differences between PTSD cases and controls.

Our study included a cohort of 12 subjects with half healthy controls. The Hurst exponent of the PTSD subjects is found to be significantly smaller than the healthy controls in channel F3. Our results indicate that F3 may be a useful channel for diagnostic applications of Hurst exponents in distinguishing PTSD and healthy subjects.

*Keywords:* Hurst exponent, EEG, Stationary.

**Introduction**

EEG (Electroencephalogram) signal measures voltage temporal variations, which reflects brain neuronal electrical activity^^[[1]](#endnote-1)^^. The EEG signals contain relevant dynamic information about the brain’s electrophysiological activity. Thus, prediction and modeling EEG signals is an important area of biological and biomedical research.^^[[2]](#endnote-2)^,^[[3]](#endnote-3)^^ EEG signals feature non-linear and non-stationary pseudo oscillatory behavior characterizing spontaneous brain oscillations such as alpha waves. To extract important features of EEG for the diagnosis of different diseases, advanced signal processing techniques are required. There are various states and conditions that influence the signals - such as sleep, epilepsy, reflexology, drugs/anesthesia, diabetes, meditation, experiencing emotions, listening to music - as well as artifacts that influence the signals.^^[[4]](#endnote-4)^^ Long-term and short-term characteristics of EEG time series have been investigated in biological applications,^^[[5]](#endnote-5)^^ and EEG time series have been studied to identify affected regions of the brain in disease, such as epilepsy.^^[[6]](#endnote-6)^^

In the current study, EEG was employed to study time-series differences related to post-traumatic stress disorder (PTSD). In a study of the dynamical complexity of EEG time series in 27 PTSD and 14 healthy people, Jeong-Ho Chae et al. (2004) found reduced complexity in channels Fp1, F8, C4, P4, T3, T4, T5, T6 and O1 for PTSD cases.^^[[7]](#endnote-7)^^ Another group calculated non-linear independence (NI) values of EEG data of 16 channels corresponding to 18 pairs of PTSD and healthy controls. They showed that, in PTSD patients, NI factors increase in channels F3, F7, C3, T5, P3 and decrease in channels F4, C4, P4, and O2.^^[[8]](#endnote-8)^^ In five case studies, Rutter (2014) determined channels F3, F4, C3, C4, P3, P4, Fz, Cz, and Pz as the most influenced ones by the disorder^^[[9]](#endnote-9)^^.

There have been several studies on the application of the Hurst exponent to investigate EEG signals^^[[10]](#endnote-10)^^. The Hurst exponent is a measure of the long-memory properties of signals.^^[[11]](#endnote-11)^,^[[12]](#endnote-12)^^ In this study, we aim to explore the possibility of developing a Hurst exponent-based method for feature selection of channels that may be important for prediction. We hypothesize that the long memory of the EEG signals in the PTSD and healthy controls groups differentiateeach other. To this end, we investigate the long-memory properties of the EEG data by applying the time dependent Hurst analysis using the rescaled range (R/S) technique.

The manuscript is organized as follow. First, the EEG data are described statistically. Next, the theoretical approach of the Hurst exponent calculation including the R/S analysis method and the importance of stationary data are explained. Finally, the results are presented and discussed.

**Material and Methods**

EEG data were collected at the Laureate Institute for Brain Research as part of a simultaneous EEG and fMRI study^[[13]](#endnote-13)^ conducted on individuals with combat-related PTSD and healthy controls. The study was approved by the Western Institutional Review Board, Puyallup, WA. All procedures with human subjects were conducted according to the code of ethics of the World Medical Association (Declaration of Helsinki) for experiments involving humans. All subjects gave written informed consent to participate in the study and received financial compensation.

**1. Data description**

Six PTSD individuals and six healthy controls (mean age = 27 ± 5 years, all male) were involved in this study. For each subject, EEG signals from 31 channels (Fp1, Fp2, F3, F4, C3, C4, P3, P4, O1, O2, F7, F8, T7, T8, P7, P8, Fz, Cz, Pz, Oz, FC1, FC2, CP1, CP2, FC5, FC6, CP5, CP6, TP9, TP10, POz) were recorded with the ground and reference electrodes positioned at AFz and FCz. One channel was placed at subject’s back to measure electrocardiogram. The EEG signals were recorded at a sampling rate of 5000 samples/s and a resolution of 0.1μV. The EEG preprocessing was carried out in the proprietary software BrainVision Analyzer2 (Brain Products, GmbH). For further analysis we used EEGLAB software (<http://sccn.ucsd.edu/eeglab>^Ref.a^). The original data is attached in supplementary materials.

For the EEG preprocessing, MRI gradient artifact and cardioballistic (BCG) artifact were removed using the template subtraction method^17,18^. After the gradient artifact removal, the EEG data was down sampled to 250 samples/s (4 ms temporal resolution) and low-pass filtered to 40Hz. Residual cardio ballistic artifact, as well as blink and saccade artifacts, were removed using independent component analysis (ICA). Due to motion of PTSD subjects during the fMRI scan, we removed time periods with subject head motion. In the experiment, the scan lasted for 526 s. The first 6s was removed for steady-state signals. There were 130,000 time points in each channel. For the analysis, we included only 50,000 data points by selecting the first available 50,000 points without subject motion. Provided that there are sufficient EEG data points to reach stationarity, using fewer data points does not affect the results statistically but decreases the calculation time.

For the Hurst analysis, we calculated the temporal changes in the preprocessed data. As we will discuss later in Section 2.2, the Hurst exponent differentiates most strongly between healthy and PTSD subjects for the F3 channel. Thus, we summarize the statistics of the F3 channel data for all subjects (Table 1). Note that positive skewness and kurtosis of the EEG data are found for both groups of subjects. The positive skewness indicates the asymmetrical distribution of the EEG signal amplitude with a long tail to the right. Furthermore, the positive kurtosis suggests that the distribution about the mean is more peaked than a Gaussian distribution. EEG time-series distributions in µV for channel F3 for each subject are shown in Fig 1. The distribution of the other channels is given in supplementary materials, Appendix I.


**Table 1**: Summary of EEG statistics for PTSD and Healthy subjects for channel F3.

| **Subject** | **Mean** | **Std.** | **Skewness** | **Kurtosis** |
| --- | --- | --- | --- | --- |
| **Healthy** | 28.9012 | 5.9296 | 0.0737 | 2.5290 |
| **PTSD** | 32.0050 | 2.6315 | 0.0398 | 3.1504 |

**Fig 1: EEG time-series distributions in µV for channel F3 for each subject.** The red box plots represent PTSD and green box plots represent healthy controls.

**2. Theoretical approach**

**2.1. R/S analysis**

R/S method was employed to estimate the Hurst exponent of time series as a measure of the long-range correlation^[[14]](#endnote-14)^. This method can be described by the following steps:

*Step 1*: Calculate the logarithmic retunes of detrended time series with length $N=r-1$, where $t$ has length of original time series.

$N_{i}=log\left( \frac{t_{i+1}}{t_{i}} \right) i=1,2, \cdots,r-1$ (1)

*Step 2*: Split the time series into $m$ adjoining subsets $S_{j}$ of length$n$, where $m\times n=N$, and $j=1,2,\cdots,m.$ The segments of each subset calls $N_{k,j}$, with $k=1,2,\cdots,n.$ The average of each subset $S_{j}$ is counted by:

$M_{j}=\frac{1}{n}\sum_{k=1}^{n} \sum_{j=1}^{m} N_{k,j}M_{j}=\frac{1}{n}\sum_{k=1}^{u} N_{k,j}$ (2)

*Step 3:* Calculate the addition of deviation from the average for each subset of $S_{j}$ as:

$X_{k,j}=\sum_{i=1}^{k} \sum_{j=1}^{m} {(N}_{i,j}-M_{j}), k=1,2,\cdots,n X_{k,j}=\sum_{i=1}^{k} {(N}_{i,j}-M_{j}), k=1,2,\cdots,n$ (3)

*Step 4:* The mean relative range of any single subset which surf the time series is calculated as:

$R_{I_{j}}=\max\left( X_{k,j} \right)-\min\left( X_{k,j} \right), 1<k<n$ (4)

*Step 5:* In this step, standard deviation of each subgroup is considered:

$S_{I_{j}}=\sqrt{\frac{1}{n}\sum_{k=1}^{n} {\sum_{j=1}^{m} \left( N_{k,j}-M_{j} \right)}^{2}.}S_{I_{j}}=\sqrt{\frac{1}{n}\sum_{k=1}^{n} \left( N_{k,j}-M_{j}^{2} \right).}$ (5)

*Step 6:* The range $R_{I_{j}}$ of each subset rescaled by the related standard deviation $S_{I_{j}}$. Therefore, the average $R/S$ measures for each window with length $n$ is:

${(R/S)}_{n}=\frac{1}{m}\sum_{j=1}^{m} \left( \frac{R_{I_{j}}}{S_{I_{j}}} \right).$ (6)

All above steps should be repeated for different time periods.

*Step 7:* Plot $log{(R/S)}_{n}$ versus $log(n)$. The slope of this graph shows the Hurst exponents H. ^[[15]](#endnote-15)^

Hurst values could be calculated using Rescaled range formula estimated by above steps.

$RR=(2^{\left( 2H-1 \right)}-1)\times n^{H}$ (7)

Where $H$is the Hurst exponent for each EEG signal and $n$ is the number of data points^[[16]](#endnote-16),^^[[17]](#endnote-17),^^[[18]](#endnote-18)^

**2.2. Stationarity of data**

A time series is considered stationary when its statistical properties such as mean, variance, autocorrelation, etc., are constant over time. In terms of the probability, if the probability distribution function of a time series does not change with time, it can be considered as a stationary process.^[[19]](#endnote-19),^^[[20]](#endnote-20)^ In practice, most of statistical forecasting methods are based on the assumption that the time series can be rendered approximately stationary through the use of mathematical transformations.

The R/S method estimates reliable Hurst exponents only for stationary time series while EEG signals present strong non-stationary characteristics.^[[21]](#endnote-21)^ Thus, to investigate the dynamical Hurst exponents of EEG signals, the issue of non-stationarity of data should be resolved.^[[22]](#endnote-22)^ To this end, one possibility is to process the data within a window that is large enough so that the data statistically behave like a stationary time series. This approach would be beneficial only if the statistical properties of data such as mean, standard deviation, etc. saturate over an increasing time scale.

In this study, we used the variation of the standard deviation calculated within different time window lengths to estimate the window width that best fulfills the stationary criterion. The stationary criterion of different channels is separately calculated and may be different from each other. Since the Hurst exponent calculation for each channel in one subject was time consuming, we performed a preliminary data examination using a smaller set of subjects (first available eight subjects) to determine which electrodes to be focused on for further analysis. In the preliminary analysis, we calculated the time variations of the standard deviations and the Hurst exponents for all channels (Appendices II-III in the supplementary material). Since the Hurst exponents for all channels, except F3, did not show any significant group difference, we focus on channel F3 for the Hurst exponent calculations and further analysis. The standard deviation of F3 against different time window length for all subjects is shown in Fig. 2.

**Fig 2: Standard deviation of the EEG data against length of the time window: 6 PTSD subjects (orange) and 6 healthy controls (blue) of channel F3. 3. Results and discussions**

Positive skewness and kurtosis indicate deviation from a Gaussian distribution. Our statistical inferences demonstrate that the EEG data are strongly non-Gaussian (Table 1). To prepare the data for the estimation of the Hurst exponent, the data are segmented according to the saturation window length as explained in Section 2.2. The saturation window length or, as we call it, the stationary point for each EEG signal is determined by calculating the signal standard deviation versus time for all 31 channels of all twelve subjects.

We compute the variation of the standard deviation over time for channel F3 for each of the 12 subjects (Fig. 2). Each curve corresponds to a healthy or PTSD subject with 49 windows each with 1000 data. The closest power of 2 for the stationary point is plotted in dotted line. Our results show that, although for many EEG signals the standard deviation saturates over a few thousand data points, the largest saturation point that is large enough for both the original and filtered data to be considered stationary is 32,768 (or 131 second).

Once we determined the window length within which the EEG data can be considered stationary (32,768 points), we then perform the Hurst exponent calculations within moving windows of this length for all EEG channels and subjects. The moving window is defined in such a way that the window of data slides over the time series each iteration with the original beginning 1,000 data points removed and the next 1,000 new data points updated at the end of the window for the 50,000 data points considered in each EEG channel, there are almost 17,000 moving windows, and hence, 17,000 Hurst exponents.

The Hurst exponents calculated for the representative channel F3 from the preprocessed data are presented in Fig 3. The readers may find the Hurst exponents for other channels in the supplementary material, Appendix III. Fig. 3 shows that all subjects, healthy and PTSD, possess Hurst exponents with highly persistent behavior (H > 0.5). The high Hurst exponent values are indicative of the existence of strong correlation in the data, which leads to long-term memory of the data. The Hurst exponent separation between healthy and PTSD subjects is small for channel F3, but the difference between the groups is statistically significant (Fig 3). We used a Mann-Whitney U test to investigate the null hypothesis of no difference in the Hurst exponent between PTSD and control groups. The Hurst exponent of the PTSD group is found to be significantly smaller than the healthy controls. (p < 0.0260) (Table 2).


**Table 2**: Hurst exponents for the healthy and PTSD subjects for F3 channel, and the p-value calculated by Mann-Whitney U test for the difference of the Hurst exponents between two groups.

|  | H1 | H2 | H3 | H4 | H5 | H6 | P1 | P2 | P3 | P4 | P5 | P6 |
| --- | --- | --- | --- | --- | --- | --- | --- | --- | --- | --- | --- | --- |
| Average of Hurst exponent | 0.9505 | 0.9436 | 0.9522 | 0.9807 | 0.9640 | 0.9520 | 0.9357 | 0.9170 | 0.9103 | 0.9118 | 0.9591 | 0.9064 |
| Std.dev.of Hurst exponent | 0.0041 | 0.0115 | 0.0159 | 0.0076 | 0.0038 | 0.0061 | 0.0069 | 0.0094 | 0.0079 | 0.0084 | 0.0262 | 0.0186 |
| Group average | 0.9572 (S.D. = 0.0133) | | | | | | 0.9234 (S.D. = 0.0203) | | | | | |
| Group difference | 0.0338 with p-value = 0.0260 | | | | | | | | | | | |

**Fig 3: Hurst exponent distribution:** 6 Healthy controls (green box) and 6 PTSD cases (red box) of channel F3

Our findings suggest that the F3 channel discriminates between PTSD and healthy controls based on the Hurst exponent. The relevance of channel F3 to PTSD is consistent with other reports [8,9]. Non-linear independence (NI) values of PTSD and healthy controls calculated by J. Kim and collaborators show that in PTSD patients NI factors increases in channel F3.^8^ In five case studies, Rutter (2014) determined F3 as one of the most associated channels with the disorders.^9^

Hurst exponent analyzes the long term memory and data dependency. In addition to the potential diagnostic insights of the Hurst values, it also uses more information from the dataset, which provides more stable estimates.

F3 is located in the frontal region of brain, which is related to emotion recognition responsibilities. Furthermore, it involves the tasks of judgment, planning, and sustained attention, inhibition of responses, verbal episodic memory retrieval, and problem solving, sequencing, and deducing facts to conclusions. Changes in the EEG alpha band have been investigated in multiple studies.^^[[23]](#endnote-23)^^[[24]](#endnote-24)^,^[[25]](#endnote-25)^,^[[26]](#endnote-26)^^; however, we did not find a significant difference between PTSD and healthy subjects in the Hurst exponent for the EEG alpha band.

**Acknowledgments.**

The authors would like to acknowledge Bill White for his helps in programming and Tandy supercomputing center for many hours of computing time and supports.

This research was supported in part by W81XWH-12-1-0697 grant from the US Department of Defense to JB and CKW.

**Author Contributions**

BR: conceived the ideas of the paper, analyzed the data and wrote the paper. CKW: collected, pre-processed, and analyzed the data. PN: analyzed the data and contributed in analysis tools, writing and programming. JB: provided the data and supervised the biological and statistical approaches. BM: supervised the computational and statistical approaches. All authors commented on this paper.

**Fig 1: EEG time-series distributions in µV for channel F3 for each subject**. The red box plots represent PTSD and green box plots represent healthy controls.

EEG data points (0.1µV)

Subjects

**Max saturation**

**point=2^15^**

Standard deviation

Window Length

Fig 2: Standard deviation of the EEG data against length of the time window: 6 PTSD subjects (orange) and 6 healthy controls (blue) of channel F3.


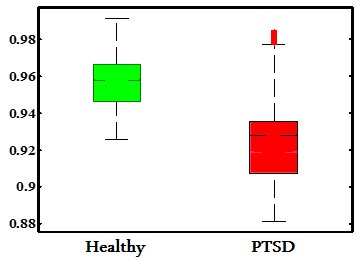


Hurst exponents

**Fig 3: Hurst exponent distribution:** 6 Healthy controls (green box) and 6 PTSD cases (red box) of channel F3

**Supporting Information**

**APPENDIX I:** Box Plot of Original Data – H: Healthy Controls, P: PTSD Cases Page 2

**APPENDIX II:** Standard Deviation of Train Dataset Including 8 Subjects to Find the Stationary Points – Blue: Healthy Controls, Red: PTSD Cases Page 6

**APPENDIX III:** Hurst Exponents of Train Dataset Including 8 Subjects– Blue: Healthy Controls, Red: PTSD Cases Page 10

1. **1. Niedermeyer E, da Silva FL**. Electroencephalography: Basic Principles, Clinical Applications, and Related Fields. Lippincot Williams & Wilkins. [ISBN](http://en.wikipedia.org/wiki/International_Standard_Book_Number), 2004; [0-7817-5126-8](http://en.wikipedia.org/wiki/Special:BookSources/0-7817-5126-8). [↑](#endnote-ref-1)
2. **. Zaremba W.** Modeling the variability of EEG/MEG data through statistical machine learning, Master’s thesis in mathematics, University of Warsaw, 2012. [↑](#endnote-ref-2)
3. **. Coben R, Evansn JR.** neurofeedback and neuromodulation techniques and applications, Elsevier, 2011. [↑](#endnote-ref-3)
4. **. Lopes da Silva F.** EEG – fMRI Physiological Basis, Technique, and Applications, Springer, 2010; 19-38. [↑](#endnote-ref-4)
5. **. Ingber L.** Statistical mechanics of neocortical interactions: Training and testing canonical momenta indicators of EEG. Mathematical Computer Modelling. Volume 27. Number 3. 1998; 33-64.  [↑](#endnote-ref-5)
6. **. Abou-Khalil B,Musilus,** Atlas of EEG & Seizure Semiology. Elsevier, 2006. [↑](#endnote-ref-6)
7. **. Chaea J, Jeongb J, B. Petersonc, Kima D, Bahka W, Juna T, et all.** Dimensional complexity of the EEG in patients with posttraumatic stress disorder, Psychiatry Research: Neuroimaging Volume 131, Issue 1, 30 May 2004;79–89. [↑](#endnote-ref-7)
8. **. Kim J, Chae J, Ko H, Latchoumane CV,Banerjee A, Mandell DJ, et all.** Hemispheric asymmetry in non-linear interdependence of EEG in post-traumatic stress disorder, Psychiatry and Clinical Neurosciences 2012; 66: 87–96. [↑](#endnote-ref-8)
9. **. Rutter P.** Five case studies using live z- score training percent-z ok on individuals diagnosed with PTSD, NeoroConnections, 2012. [↑](#endnote-ref-9)
10. **. Subha DP, Joseph PK,Acharya R, Lim CM.** EEG Signal Analysis: A Survey, J Med Syst , 2010;34:195–212. [↑](#endnote-ref-10)
11. **. Hurst HE, Black RP, Simaika YM.** Long-term storage: an experimental study. London: Constable, 1965. [↑](#endnote-ref-11)
12. **. Hurst HE.** Long term storage capacity of reservoirs. Trans. Am. Soc. Eng. 1951;116: 770–799. [↑](#endnote-ref-12)
13. **. Mulert C, Lemieux L.** EEG-fMRI: Physiological Basis, Technique, and Applications. Springer-Verlag, Berlin Heidelberg, Eds., 2010. [↑](#endnote-ref-13)
14. **.** **Fan J.** Rescaled Range Analysis in Higher Dimensions, Research Journal of Applied Sciences, Engineering and Technology 5(18): 4489-4492, 2013. [↑](#endnote-ref-14)
15. . **Jokic-Begic N, Begic D,** Quantitative electroencephalogram (qEEG) in combat veterans with post-traumatic stress disorder (PTSD). Nord J Psychiatry. 2003;57(5):351–355. [↑](#endnote-ref-15)
16. **. Norouzzadeh P, Jafari GR.** Application of multifractal measures to Tehran price index, Physica A, 2005, Volume 356, 609–627. [↑](#endnote-ref-16)
17. **. Voss J.** Rescaled Range Analysis: A Method for Detecting Persistence, Randomness, or Mean Reversion in Financial Markets, CFA institute, 2013. [↑](#endnote-ref-17)
18. **. Rose O.** Estimation of the Hurst Parameter of Long Range Dependent Time Series, University of Wurzburg, Research Report Series, 1996. [↑](#endnote-ref-18)
19. **. Nason GP.** Wavelet Methods in Statistics with R, Springer, Chapter 11, 2008. [↑](#endnote-ref-19)
20. **. Norouzzadeh P, Rahmani B, Norouzzadeh** MS [Forecasting smoothed non-stationary time series using genetic algorithms](http://www.worldscientific.com/doi/abs/10.1142/S0129183107011133), International Journal of Modern Physics C, 2007. [↑](#endnote-ref-20)
21. **. Korolev VY, Skvortsova NN.** Stochastic Models of Structural Plasma Turbulence, Walter de Gruyter, 2006. [↑](#endnote-ref-21)
22. **.** [**Morales**](http://arxiv.org/find/q-fin/1/au:+Morales_R/0/1/0/all/0/1) **R,**[**Di Matteo**](http://arxiv.org/find/q-fin/1/au:+Matteo_T/0/1/0/all/0/1) **T, [Gramatica](http://arxiv.org/find/q-fin/1/au:+Gramatica_R/0/1/0/all/0/1) R, [Aste](http://arxiv.org/find/q-fin/1/au:+Aste_T/0/1/0/all/0/1) T.** Dynamical Hurst exponent as a tool to monitor unstable periods in financial time series, arXiv:1212.3195v2. 2013. [↑](#endnote-ref-22)
23. **.Wahbeh H, Oken BS.** Peak High-Frequency HRV and Peak Alpha Frequency Higher in PTSD, Appl Psychophysiol Biofeedback. 2013 Mar;38(1): 57–69. [↑](#endnote-ref-23)
24. **. Rabe S, Beauducel A, Zollner T, Maercker A, Karl A,** Regional brain electrical activity in posttraumatic stress disorder after motor vehicle accident. J Abnorm Psychol. 2006;115(4):687–698. [↑](#endnote-ref-24)
25. **. Jokic-Begic N, Begic D,** Quantitative electroencephalogram (qEEG) in combat veterans with post-traumatic stress disorder (PTSD). Nord J Psychiatry. 2003;57(5):351–355. [↑](#endnote-ref-25)
26. **. Metzger LJ, Paige SR, Carson MA, et al.** PTSD arousal and depression symptoms associated with increased right-sided parietal EEG asymmetry. J Abnorm Psychol. 2004;113(2): 324–329. [↑](#endnote-ref-26)
